# Antiviral Activity of Brazilian Propolis from Stingless Bees Against Rotavirus

**DOI:** 10.3390/microorganisms13061424

**Published:** 2025-06-19

**Authors:** Guadalupe González-Ochoa, Ana Paola Balderrama-Carmona, Jesús Antonio Erro-Carvajal, José Guadalupe Soñanez-Organis, Luis Alberto Zamora-Álvarez, Marcelo Andrés Umsza Guez

**Affiliations:** 1 Laboratorio de Microbiología e Inmunología, Departamento de Ciencias Químico-Biológicas y Agropecuarias, Facultad Interdisciplinaria de Ciencias Biológicas y de la Salud, Universidad de Sonora, Navojoa 85880, Mexico; guadalupe.gonzalezochoa@unison.mx (G.G.-O.); a214200126@unison.mx (J.A.E.-C.); jose.organis@unison.mx (J.G.S.-O.); luis.zamora@unison.mx (L.A.Z.-Á.); 2 Departamento de Biotecnologia, Instituto de Ciências da Saúde, Universida de Federal da Bahia, Salvador 40110-909, Brazil

**Keywords:** propolis, phenolic compounds, rotavirus, gastroenteritis

## Abstract

Group A rotavirus (RV) causes gastrointestinal disease in infants worldwide, and there is currently no specific treatment to eliminate the virus. Due to its chemical properties, propolis is a promising compound for improving gastrointestinal infections. This study aimed to evaluate the action of stingless bee propolis against RV. The method involved determining the concentrations of the extracts that do not exhibit cytotoxicity in colon adenocarcinoma cells using the MTT assay and measuring the reduction in infectivity through a focus forming assay. The results showed that stingless bee propolis was non-cytotoxic up to 200 µg/mL. The reduction in RV infectivity exceeded 99% when using propolis from *Plebeia droryana* and *Melipona quadrifasciata*. Brazilian stingless bee propolis, whose active components are known for their activity against various viruses, was experimentally tested and demonstrated effective antiviral activity against RV, supporting its potential application as an antiviral agent.

## 1. Introduction

Propolis has been used for its therapeutic properties since ancient civilizations [1]. It is a plant-derived resin produced mainly by *Apis mellifera* bees to protect the hive from predators and harsh environmental conditions [2]. Bees of the Meliponini tribe, known as stingless bees (SBs) [3], are found exclusively in tropical and sub-tropical regions [4], *A. mellifera*, unlike other species, has a global distribution [5]. Propolis produced by stingless bees is also referred to as cerumen [6]. Some bee species mix propolis with soil, increasing its mineral and microbiological content [7], and this mixture is known as geopropolis [8]. Despite these differences, both cerumen and geopropolis exhibit similar beneficial effects on human health [3,9].

Propolis consists of resins, wax, pollen, vitamins, minerals, essential oils, and phenolic compounds [10]. Over 800 phenolic constituents have been identified [11]. The most biologically active compounds include flavonoids such as quercetin, galangin, kaempferol, pinocembrin, luteolin, naringenin, hesperidin, formononetin, and pinobanksin; phenolic acids such as gallic acid, caffeic acid, and coumaric acid; and stilbenes [12]. It may also contain amino acids and saponins [1].

Propolis’ biological activity as an antioxidant, anti-inflammatory, antiproliferative, antibacterial, and antiviral agent is associated with its components [13,14,15]. In this context, the medicinal and therapeutic potential of propolis has been described in the treatment of various chronic conditions, including diabetes, neurodegenerative disorders, respiratory and cardiovascular diseases, gynecological problems, dermatological conditions, and gastrointestinal disorders [1].

Gastrointestinal illness (GI) is a leading cause of death among children, the elderly, and immunosuppressed individuals worldwide [16]. It is estimated that 3 to 5 billion cases of GI disease occur globally each year [17], with approximately 70 million infant deaths annually linked to GI conditions [18]. The main viral agents responsible for GI infections include norovirus, adenovirus, astrovirus, and rotavirus (RV) [19]. RV alone causes around 258 million infections in children annually and accounts for up to 215,000 deaths in children under five years of age [20]. More than half of these deaths occur in Pakistan, India, the Democratic Republic of Congo, Nigeria, and Angola [21], although industrialized countries are also affected by GI viruses [22].

Rotaviruses (RVs) are double-stranded RNA viruses that lack a viral envelope [23]. They are transmitted via the fecal–oral route and can remain viable for several days at room temperature, showing resistance to environmental factors such as pH, temperature, and desiccation [24]. This resilience is attributed to their icosahedral structure, composed of three layers of viral proteins [25]. The virus replicates in the enterocytes of the small intestine, with an incubation period of 24 to 48 h. Symptoms—such as vomiting, fever, and diarrhea—typically last from five to seven days and can lead to rapid dehydration [26,27]. Approved vaccines for RV prevention include RotaTeq, Rotarix, Rotavac, and Rotassil [21,28]. A commonly used drug to treat RV gastroenteritis is racecadotril [29], which helps alleviate symptoms. Homeopathic alternatives, such as probiotics, also support treatment [30]. However, there is currently no FDA-approved antiviral drug specifically for RV infection [28].

Propolis has demonstrated antiviral activity against several viruses, including herpes simplex virus [31], adenovirus type 2, SARS-CoV-2 [32], vesicular stomatitis virus [33], and poliovirus types 1 and 2 [34]. Therefore, propolis produced by stingless bees (SBs) may represent a potential alternative against RV gastroenteritis due to its content of phenolic acids and tannins. This type of propolis has also shown antiviral activity against herpes simplex virus and certain arboviruses [35]. 

The antiviral properties of propolis are attributed to compounds such as kaempferol, which inhibits SARS-CoV-2 infectivity by blocking viral fusion with cell membranes [32]; caffeic acid, which interferes with hepatitis C virus replication [33]; and formononetin, which reduces the expression of viral protein 1 in enterovirus 71 (EV71), thereby decreasing the number of viral particles [34]. However, antiviral activity of propolis against RV has not been reported to date.

Given the significant health burden of RV-related gastrointestinal disease, its high mortality rate in infants worldwide, and the absence of specific treatments, this study aimed to evaluate the antiviral activity of Brazilian propolis against RV.

## 2. Materials and Methods

### 2.1. Origin and Extractions of the Propolis Samples

The propolis samples were donated by the Food Science Postgraduate Program at the Faculty of Pharmacy, Federal University of Bahia (UFBA), under the supervision of Dr. Marcelo Andres Umsza Guez, and were previously analyzed in a study by Rocha et al. [36]. The samples were collected in the state of Bahia, Brazil, and are thus labeled as Brazilian propolis (PB). Propolis samples with the highest phenolic content from stingless bee species were selected—*Plebeia droryana* (PB1 and PB3), *Frieseomelitta doederleini* (PB2), and *Melipona quadrifasciata* (PB4 and PB5) [36]. It is important to emphasize that the propolis samples whose phenolic content was characterized in previous studies are the exact same specimens used in the present investigation to evaluate antiviral activity.

Ethanolic extracts of propolis (EEP) were obtained following the method described by Escriche and Borras (2018), with modifications [37]. Under constant agitation, 15 mL of 80% ethanol was added to 2 g of raw propolis for 1 h. After this, the mixture was centrifuged at 8800 rpm for 11 min at 5 °C. The supernatant was filtered using Whatman No. 1 paper and then dried in a laboratory oven (Fisher Scientific, Hampton, NH, USA) at 45 °C for 3 days. The samples were wrapped in aluminum foil and stored at −20 °C until analysis.

### 2.2. Cell Lines

Epithelial kidney cells derived from the African Green Monkey (Cercopithecus aethiops, MA-104, ATCC CRL-2378) were used for RV replication and titration. Colon adenocarcinoma cells (HT-29, ATCC HTB-38) were used to test propolis antiviral activity assays. The MA-104 and HT-29 cell lines were selected based on their widespread acceptance and extensive use as in vitro models for human rotavirus infection. These cell lines have been well validated in the literature for replicating key aspects of the viral life cycle and host–pathogen interactions in the gastrointestinal context [38]. Consequently, the inclusion of additional cellular models was not considered necessary for the scope and objectives of the present study. The cell lines were incubated following the method described by Romero-Arguelles et al. with modifications [39]. In 2 mL of an RPMI cell culture medium (Gibco^®^, 1640, Billings, MT, USA), supplemented with 10% fetal bovine serum (FBS, Biowest, Nuaillé, France) and 1% antifungals and antibiotics (Caisson^®^, North Logan, UT, USA), the cell suspension was transferred to a T-25 flask (Corning^®^, Corning, NY, USA). An additional 3 mL of the RPMI cell medium was then added. The suspension was incubated at 37 °C with 5% CO_2_ until confluence.

### 2.3. Cell Viability Assay

For cell viability, the Bromide 3-[4,5-dimethylthiazol-2-yl]-2,5-diphenyltetrazolium (MTT) test was used [38]. One milligram of EEP was added to 10 µL of dimethyl sulfoxide (DMSO) (Sigma-Aldrich, St. Louis, MO, USA) and 990 µL of RPMI medium. Serial dilutions were performed to obtain final 100, 200, 400, and 800 µg/mL concentrations. In a 96-well microplate, a cell subculture was prepared with 100 µL of RPMI supplemented with 7% FBS, with a confluence of 2 × 10^5^ HT-29 cells/mL and incubated for 24 h at 37 °C with 5% CO_2_.

Two washes were performed with 0.9% saline to add 100 µL of each EEP dilution and 100 µL of RPMI medium supplemented with 7% FBS, resulting in a 1:2 dilution in each well. The final concentrations of EEP were 50, 100, 200, and 400 µg/mL. The plates were incubated 24 h 37 °C with 5% CO_2_. Subsequently, two washes were performed again, and 20 µL of MTT at a concentration of 5 g/mL was added, followed by incubation for 3 h. Next, 100 μL of DMSO was added to each well for formazan dissolution, and absorbance was measured at 570 nm. The results of this test were interpreted using the ISO-10993-5:2009 procedure [39].

### 2.4. Quantification of the Inicial Concentration of the Wa Rotavirus Strains

Susana López Charreton from the Biotechnology Institute (IBT) at the Autonomous University of Mexico provided the RV Wa used in this study. The focus forming assay (FFA) was quantified using the microtitration technique described by Chasey [40]. A subculture was prepared in a 96-well microplate with 150 μL of RPMI supplemented with 7% FBS, containing 2 × 10^5^ MA-104 cells/mL in each well, and incubated for 48 h. Separately, 350 μL of RV (to estimate a multiplicity of infection of 1) was activated using 5 μL of trypsin (Sigma-Aldrich) and incubated at 37 °C with 5% CO_2_ for 30 min. Subsequently, serial dilutions of the trypsinized RV-A in RPMI without FBS were prepared at ratios of 1:2, 1:4, 1:8, 1:16, 1:32, and 1:64. Fifty microliters of each RV dilution were added to the wells containing MA-104 cells and incubated for 1 h.

After the incubation period, the cells were removed from the culture medium, the monolayer was washed with 200 μL of PBS, and 105 μL of RPMI without FBS was added to each well and incubated for 14 h at 37 °C with 5% CO_2_.

Afterward, the cells were removed from the culture medium, the monolayer was washed with PBS, and the cells were fixed with 100 μL of an 80% PBS-acetone solution for 40 min at room temperature. The cells were washed with PBS and incubated with 75 μL of anti-rotavirus antibody (Invitrogen, Waltham, MA, USA), diluted in a 1:1000 PBS ratio. Following this, the cells were incubated with 75 μL of the conjugated antibody horseradish peroxidase (HRP)-anti-IgG (Invitrogen), diluted at 1:800 in PBS, in each well and incubated for 1 h. The monolayer was washed and incubated for 15 min at 37 °C with 75 μL of carbazole substrate (8 mL of 0.05 M acetate buffer, 20 μL of 30% H_2_O_2_, and 3 mL of carbazole solution). Subsequently, the plate was submerged in tap water five times for 30 s each and allowed to dry. The infected cells were counted using an optical microscope. The FFA count was calculated using the following formula: FFA/mL =$\;\left(number\;of\;foci\right)\left(20\right)\;(dilution)$. RV multiplicity of infection (MOI) was calculated by dividing the FFA/mL by the cell confluence seeded in each well.

### 2.5. Antiviral Activity of Brazilian EEP Against RV Strain

In a 6-well plate with HT-29 cells at 95% confluence, 115 µL of viral lysate was added to achieve a multiplicity of infection (MOI) of 0.05 and incubated for 1 h. Following this, EEP was added to RV-infected cells and incubated for 24 h. The cells were then subjected to three cycles of freezing and thawing. The lysates obtained were stored and used in the microtitration assay to determine the viral load. To ensure the specificity of the observed antiviral activity, appropriate negative controls were included in all assays. Virus-infected cells without treatment served as the baseline for maximal viral replication, while an additional control consisting of virus-infected cells treated with the vehicle (DMSO) at the same concentration used for extract dilution was included to assess potential solvent effects. Neither control condition resulted in a significant reduction in viral replication, confirming that the detected antiviral activity was specifically attributable to the bioactive components of the tested extracts.

### 2.6. Statistical Analysis

The samples were analyzed in triplicate. Central tendency analysis, as well as ANOVA and Tukey’s post hoc tests, were performed to evaluate differences between groups. Statistical significance was considered at *p* < 0.05. Graphs were generated using GraphPad Prism version 10 (GraphPad Software Inc., La Jolla, CA, USA).

## 3. Results

The obtained ethanolic extracts of propolis (EEPs) showed the following recovery percentages from raw propolis: 30% for PB1, 20% for PB2, 20% for PB3, 10% for PB4, and 20% for PB5. Viability assays were performed using diluted EEPs at different concentrations. A reduction in cell viability to 50% or less was observed at concentrations of 100 μg/mL, 200 μg/mL (except for PB4), and 400 μg/mL compared to other concentrations.

**Figure 1:**
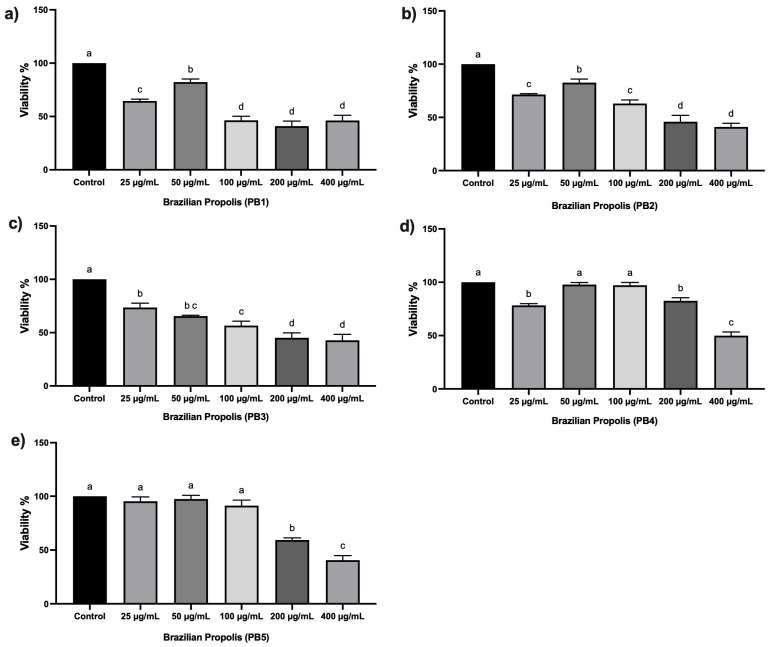
Percentage of cellular viability of HT-29 cells treated with EEP at different concentrations (25, 50, 100, 200, and 400 µg/mL). (**a**) PB1, (**b**) PB2, (**c**) PB3, (**d**) PB4, and (**e**) PB5. HT-29 cell without EEP was used as the control. Letter variety indicates statistical significance (*p*-value < 0.05).

MTT assays were conducted to determine the concentrations of propolis extract that do not produce toxic effects on the HT-29 cancer cell line. These non-cytotoxic concentrations were subsequently selected for antiviral assays against rotavirus to ensure that any observed antiviral activity could be attributed to the extract itself and not to a loss of cell viability. The cells treated with PB1 showed a viability of 64% (±4) at a 25 μg/mL, and 82% (±5) at 50 µg/mL. However, at higher concentrations (100 µg/mL, 200 µg/mL, and 400 µg/mL) cell viability decreased to 46.4% (±4), 40.9 % (±5), and 46.2% (±5), respectively. The cellular viability for PB2 was 71% (±3) at 25 μg/mL, 82% (±4) μg/mL at 50 µg/mL, 63% (±6) at 100 µg/mL, 46% (±6) at 200 µg/mL, and 41% (±4) at 400 µg/mL. Treatment with PB3, resulted in a cellular viability of 73% (±5) in cells treated with the concentration of 25 μg/mL, 65% (±3) with 50 μg/mL, 54% (±5) with 100 μg/mL, and less than 45% with 200 μg/mL and 400 μg/mL. The cellular viability of cells treated with PB4 was from 80% (±2) to 98% (±4) with the concentrations 25 μg/mL, 50 μg/mL, 100 μg/mL, and 200 μg/mL, but it dropped to 50% (±5) with the concentration of 400 μg/mL. Finally, with PB5 treatment, the cellular viability ranged from 91% (±5) to 97% (±5) with concentrations of 25 μg/mL, 50 μg/mL, and 100 μg/mL; it decreased to 60% (±4) and 40% with the concentrations of 200 μg/mL and 400 μg/mL, respectively (Figure 1). The concentration associated with a higher cell viability percentage of each PB treatment was chosen to study the antiviral effect of EEP. To further determine the anti-RV activity of PB1, PB2, and PB3 in other assays, the concentration in each assay was 50 µg/mL; in PB4, the concentration was 200 µg/mL, and in PB5, the concentration was 100 µg/mL.

The effect of EEP against RV was tested, with RV propagated in MA-104 cells. The FFU assay was selected as the primary method for evaluating antiviral activity because it enables direct visualization of viral spread in cell cultures. This technique is particularly valuable for detecting localized cytopathic effects, allowing for a more accurate assessment of how the antiviral compound influences viral infectivity and cell-to-cell transmission. Additionally, the FFU assay provides reliable quantification of infectious viral particles, making it a suitable and informative method for the objectives of this study [41].

**Figure 2:**
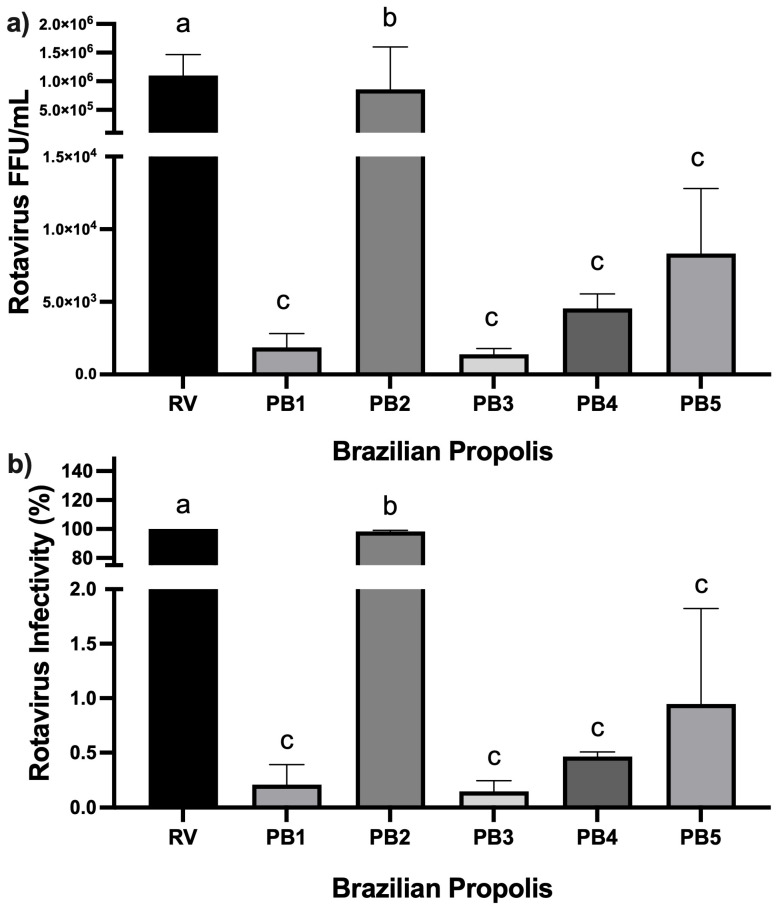
Effect of Brazilian propolis on rotavirus (RV) infection. (**a**) Brazilian propolis and its effect on the RV load in FFU/mL. (**b**) Brazilian propolis and its effect in the RV infectivity on percentage. The propolis samples PB1 to PB3 were tested at 50 µg/mL; PB4 was 200 µg/mL, and PB5 was 100 µg/mL. Letter variety indicates statistical significance (*p*-value < 0.05).

Figure 2a,b show the effects of PB1–PB5 on viral load and RV infectivity, respectively. In HT-29 cells infected with RV and incubated for 24 h with EEP PB3 and EEP PB1 (in separate assays), we observed a reduction in viral load was observed, ranging from 1.4 × 10^3^ FFU/mL to 1.9 × 10^3^ FFU/mL, respectively, compared to the RV control (which had a viral load of 1.3 × 10^6^ FFU/mL) without EEP treatment post-infection. Similarly, viral infectivity in cells infected and treated with PB3 and PB1 dropped by 99.8% and 99.9%, respectively. On the other hand, in RV-infected cells treated with PB4 and PB5, the viral loads were 4.5 × 10^3^ FFU/mL and 8.3 × 10^3^ FFU/mL, representing reductions of 99.5% and 99%, respectively, in viral infectivity. Finally, in cells infected and incubated with PB2, viral titers dropped to 1.2 × 10^6^ FFU/mL, corresponding to only a 1.7% reduction in viral infection.

## 4. Discussion

In this study, three samples of propolis (PB1–PB3) and two of geopropolis (PB4 and PB5) were used. First, we evaluated the cytotoxic effect of EEP to determine the best concentration for studying antiviral activity. Our results indicated a cell viability of 82% for PB1 and PB2 at 50 µg/mL; 65% for PB3 at 50 µg/mL; and 80% to 98% for PB4 and PB5 at concentrations of 200 µg/mL and 100 µg/mL, respectively (Figure 1). EEP PB1, PB2, PB4, and PB5 were considered non-cytotoxic, whereas PB3 was slightly cytotoxic according to ISO-10993-5 (2009) [42]. Similar results were reported by Barboza et al. [43], who found that geopropolis did not present cytotoxicity in non-tumoral cell lines, although it showed a decrease in cancer cell viability. Choudhari et al. [44] reported that EEP, at concentrations up to 50 μg/mL, was not cytotoxic to HT-29 cells, observing a cell survival rate of 90%. Cytotoxicity was only observed at concentrations above 250 μg/mL.

Propolis is considered safe when used according to recommendations [1]. The recommended daily dose generally varies between 300 and 1000 mg, depending on the product’s presentation and the reason for use [45]. However, propolis also has anticoagulant and anti-platelet effects, which can increase the risk of bleeding when taken with drugs like warfarin, aspirin, and clopidogrel [46], due to its benzopyrone content. Furthermore, propolis can stimulate the immune system, which may interfere with immunosuppressants like ciclosporin and tacrolimus [47].

According to the United States Cancer Institute, extracts and compounds of natural substances with an IC_50_ ≤ 30 μg/mL do not present cytotoxic effects [48]. Thus, the IC_50_ results of Brazilian propolis extracts indicate no cytotoxic risk. This suggests that it is feasible to conduct assays to identify the anti-RV effect with the confidence that the observed effect against RV is real and not an adverse effect on the cells.

On the other hand, in this work the EPP anti-RV effect was studied. In RV-infected cells treated with the EPP’s PB1, PB2, PB3, or PB4, viral infectivity dropped to 99-99-9%. Conversely, a viral reduction and infectivity decrease against RV was not observed in cells infected and treated with PB2 (Figure 2). Previously, we reported the EEP antibacterial activity and their components [36]. These samples, the same used in this study, contain formononetin, kaempferol, gallic acid, coumarin, and resveratrol in their chemical composition, and we used this previous data to discuss antiviral activity. According to previous research in these propolis samples [36], the total phenolic content, from highest to lowest, is PB4 with 82.05 ± 2.33 mg GAE/g, PB1 with 51.90 ± 2.47 mg GAE/g, PB3 with 30.13 ± 2.31 mg GAE/g, PB5 with 29.09 ± 2.63 mg GAE/g, and PB2 with 13.45 ± 1.64 mg GAE/g.

PB2 presented the lowest antiviral reduction, and its phenolic concentration was the lowest among the group of the tested propolis samples. Even so, this propolis contained p-coumaric acid (0.19 ± 0.01 mg/L), naringenin (0.11 ± 0.01 mg/L), and quercetin (0.11 ± 0.01 mg/L), among other compounds at lower concentrations. Naringenin inhibits viral replication and is considered a promising antioxidant [49,50]. Lastly, quercetin has been shown to act against RV and is reported to modulate viral replication [51]. Regardless of the presence of these components, the dose of the phenolic compounds was insufficient to reduce RV infectivity.

Geopropolis PB4 and PB5 exhibited comparable effects in reducing infectivity, with no statistically significant difference (*p*-value ≥ 0.05. As explained in the methodology, both samples were obtained from Melipona bees and although they should have been similar, variations in chemical composition occurred. PB4 contains a high concentration of formononetin (45.4 ± 0.36 mg/L), a component proven to inhibit the replication of enterovirus EV71 [52]. It also contains quercetin (0.16 ± 0.01 mg/L), which has anti-RV activity [51].

On the other hand, PB5 also contains formononetin, although in smaller quantities (1.28 ± 0.04 mg/L). However, PB5 has a higher abundance of gallic acid (4.38 ± 0.02 mg/L) than PB4, a compound with an anti-rhinovirus effect that does not present cytotoxicity [34]. Another component in geopropolis is piceatannol (0.77 ± 0.03 mg/L), which has also been shown to have antiviral effects [53].

Our results show that geopropolis PB4 and PB5 had minimal cytotoxic effects on cells. This analysis allowed us to identify appropriate concentrations to evaluate the antiviral effect against RV. Geopropolis PB4 and PB5 share some components, and differences in the concentrations of these compounds may influence their antiviral efficacy.

PB1 and PB3 also presented similar antiviral activity (*p*-value ≥ 0.05). They are produced by SBs of the *Plebeia* species and have been shown to reduce RV activity in this study. According to Rocha et al. [36], PB1 contains gallic acid (0.04 ± 0.01 mg/L), resveratrol (0.07 ± 0.01 mg/L), formononetin (0.12 ± 0.01 mg/L), piceatannol (0.11 ± 0.01 mg/L), and epicatechin. All these compounds have antiviral activity. Although PB3 does not have the highest total phenol content, it stands out for having the highest levels of coumarin (2.34 ± 0.03 mg/L) and resveratrol (0.11 ± 0.01 mg/L) compared to the other propolis samples evaluated. Resveratrol inhibits RV replication by attenuating heat shock protein 90 (HSP90) [54]. Coumarins are potent antivirals [55], but their effect on RV has not yet been demonstrated. The mechanisms associated with the antiviral activity of PB1, PB3, PB4, and PB5 might be related to blocking RV particles in the medium after their release from infected cells, as well as the activity of EEP components (previously reported as antivirals) by inhibiting viral replication. For example, formononetin inhibits EV71 by reducing RNA and protein synthesis; however, more studies are needed to clarify the antiviral mechanisms against RV.

To improve the biological activity of propolis, more advanced extraction methodologies are advised, such as the use of pressurized liquids [56], chromatographic fractionation [57], or lyophilization [43]. The latter methods are more suitable for the *Melipona quadrifasciata* species, which produces geopropolis. However, similar phenolic content has been found between geopropolis and other propolis samples [36]. The minerals present in the soil with which propolis is mixed can influence its chemical composition, thereby affecting its bioactive profile. This may result in the presence of additional compounds with antioxidants and anti-inflammatory properties, helping to mitigate cytotoxic effects. Certain minerals can have protective or modulatory effects on cells, reducing cellular damage caused by other compounds in propolis. For instance, minerals like zinc [58] and magnesium play important roles in cellular repair and immune function, potentially contributing to the lower cytotoxicity in propolis formulations obtained from areas with mineral-rich soils [59].

In the present study, a comparison was conducted between the phenolic profile of the extracts and their antiviral activity for the identification of preliminary associations between specific phenolic compounds and the observed inhibitory potency. However, we acknowledge that further studies, including assays using purified compounds and more robust multivariate analyses, are needed to establish clear causal relationships. This approach will be adopted in future investigations to better understand the mechanisms of action and validate the compounds responsible for antiviral activity.

One of the main challenges in translating in vitro findings to in vivo or clinical contexts is the bioavailability of active compounds. Phenolic compounds can undergo significant transformations during digestion, absorption, and metabolism [60], which may affect their actual antiviral efficacy in living organisms. Another important challenge is systemic toxicity and safety, as concentrations effective in vitro are not always well tolerated in vivo [61].

In addition, the physiological microenvironment, interactions with other biomolecules, and the host immune system can influence the efficacy of these compounds [62]. Therefore, future studies should include animal models and pharmacokinetic analyses to evaluate the stability, distribution, and safety of the extracts or active compounds prior to considering clinical trials.

Although propolis has demonstrated a favorable safety profile in various traditional and experimental contexts [1,9], its use in pediatric populations remains insufficiently standardized, particularly regarding dosage and formulation [63,64]. Currently, there is no consensus on the maximum safe and effective dose in children, and considerable variability exists among commercially available preparations [65]. In the present study, the highest concentration of propolis extract that did not exhibit cytotoxic effects in vitro was 400 mg/mL. However, extrapolating this concentration to in vivo settings, especially for pediatric applications, requires rigorous toxicological and pharmacokinetic investigations. Future research should aim to establish well-defined safety margins and dosing protocols to ensure the safe therapeutic use of propolis in pediatric care.

## 5. Conclusions

To our knowledge, no other studies have investigated the anti-rotavirus (anti-RV) effects of propolis. Rotavirus primarily affects individuals in vulnerable regions with a low socio-economic status and limited access to public health services, leading to the deaths of many children worldwide. This highlights the importance of continued research on propolis, its anti-RV mechanisms, and its potential for the prevention and treatment of RV infections. Further studies are needed to explore the use of propolis as a natural alternative for treating RV infections in the future.

## Data Availability

The original contributions presented in this study are included in the article; further inquiries can be directed to the corresponding authors.

## Abbreviations

The following abbreviations are used in this manuscript:

RV	Group A rotavirus
GI	Gastrointestinal disease
SBs	Stingless bees
PB	Brazilian propolis
EEP	Ethanolic extracts of propolis
MTT	Bromide 3-[4,5-dimethylthiazol-2-yl]-2,5-diphenyltetrazolium
DMSO	Dimethyl sulfoxide
IBT	Biotechnology Institute
FFU	Focus forming units
MOI	Multiplicity of infection

## References

[1] Zullkiflee N., Taha H., Usman A. (2022). Propolis: Its Role and Efficacy in Human Health and Diseases. Molecules.

[2] Bhargava P., Mahanta D., Kaul A., Ishida Y., Terao K., Wadhwa R., Kaul S.C. (2021). Experimental Evidence for Therapeutic Potentials of Propolis. Nutrients.

[3] Popova M., Trusheva B., Bankova V. (2021). Propolis of Stingless Bees: A Phytochemist’s Guide through the Jungle of Tropical Biodiversity. Phytomedicine.

[4] Salatino A., Pereira L.R.L., Salatino M.L.F. (2019). The Emerging Market of Propolis of Stingless Bees in Tropical Countries. MOJ Food Process. Technol..

[5] Boncristiani H., Ellis J.D., Bustamante T., Graham J., Jack C., Kimmel C.B., Mortensen A., Schmehl D.R. (2021). World Honey Bee Health: The Global Distribution of Western Honey Bee (*Apis mellifera* L.) Pests and Pathogens. Bee World.

[6] Chuttong B., Lim K., Praphawilai P., Danmek K., Maitip J., Vit P., Wu M.C., Ghosh S., Jung C., Burgett M. (2023). Exploring the Functional Properties of Propolis, Geopropolis, and Cerumen, with a Special Emphasis on Their Antimicrobial Effects. Foods.

[7] Lavinas F.C., Macedo E.H.B.C., Sá G.B.L., Amaral A.C.F., Silva J.R.A., Azevedo M.M.B., Vieira B.A., Domingos T.F.S., Vermelho A.B., Carneiro C.S. (2019). Brazilian Stingless Bee Propolis and Geopropolis: Promising Sources of Biologically Active Compounds. Rev. Bras. Farmacogn..

[8] Regnier L., Salatino M.-L.F., Salatino A. (2024). Parameters of the Gross Composition of Propolis from Brazilian Meliponini. J. Apic. Res..

[9] Pereira F.A.N., Barboza J.R., Vasconcelos C.C., Lopes A.J.O., Ribeiro M.N.d.S. (2021). Use of Stingless Bee Propolis and Geopropolis against Cancer—A Literature Review of Preclinical Studies. Pharmaceuticals.

[10] Rocha V.M., Portela R.D., dos Anjos J.P., de Souza C.O., Umsza-Guez M.A. (2023). Stingless Bee Propolis: Composition, Biological Activities and Its Applications in the Food Industry. Food Prod. Process. Nutr..

[11] Kasote D., Bankova V., Viljoen A.M. (2022). Propolis: Chemical Diversity and Challenges in Quality Control. Phytochem. Rev..

[12] Anjum S.I., Ullah A., Khan K.A., Attaullah M., Khan H., Ali H., Bashir M.A., Tahir M., Ansari M.J., Ghramh H.A. (2019). Composition and Functional Properties of Propolis (Bee Glue): A Review. Saudi J. Biol. Sci..

[13] Baldomir da Cruz F., Martins D.H.N., de Freitas Ferreira J., De Oliveira Magalhães P., Silveira D., Fonseca Bazzo Y.M. (2022). Antioxidant Activity of *Apis mellifera* Bee Propolis: A Review. J. Nat. Prod. Discov..

[14] Socha R., Gałkowska D., Bugaj M., Juszczak L. (2015). Phenolic Composition and Antioxidant Activity of Propolis from Various Regions of Poland. Nat. Prod. Res..

[15] Abdullah N.A., Zullkiflee N., Zaini S.N.Z., Taha H., Hashim F., Usman A. (2020). Phytochemicals, Mineral Contents, Antioxidants, and Antimicrobial Activities of Propolis Produced by Brunei Stingless Bees Geniotrigona Thoracica, Heterotrigona Itama, and Tetrigona Binghami. Saudi J. Biol. Sci..

[16] Deac L.M. (2021). Infectious Diarrhea, a Public Health Problem in Population. Biomed. Res. Clin. Rev..

[17] Wang Y., Huang Y., Chase R.C., Li T., Ramai D., Li S., Huang X., Antwi S.O., Keaveny A.P., Pang M. (2023). Global Burden of Digestive Diseases: A Systematic Analysis of the Global Burden of Diseases Study, 1990 to 2019. Gastroenterology.

[18] Rivera-Dominguez G., Ward R. Pediatric Gastroenteritis. https://www.ncbi.nlm.nih.gov/books/NBK499939/.

[19] Shonhiwa A.M., Ntshoe G., Crisp N., Olowolagba A.J., Mbuthu V., Taylor M.B., Thomas J., Page N.A. (2020). Investigation of Two Suspected Diarrhoeal-Illness Outbreaks in Northern Cape and KwaZulu-Natal Provinces, South Africa, April–July 2013: The Role of Rotavirus. S. Afr. J. Infect. Dis..

[20] Omatola C.A., Olaniran A.O. (2022). Rotaviruses: From Pathogenesis to Disease Control—A Critical Review. Viruses.

[21] Varghese T., Kang G., Steele A.D. (2022). Understanding Rotavirus Vaccine Efficacy and Effectiveness in Countries with High Child Mortality. Vaccines.

[22] Du Y., Chen C., Zhang X., Yan D., Jiang D., Liu X., Yang M., Ding C., Lan L., Hecht R. (2022). Global Burden and Trends of Rotavirus Infection-Associated Deaths from 1990 to 2019: An Observational Trend Study. Virol. J..

[23] Caddy S., Papa G., Borodavka A., Desselberger U. (2021). Rotavirus Research: 2014–2020. Virus Res..

[24] Hatib A., Hassou N., Ennaji M. (2021). Monitoring of Group A Rotavirus Strains Circulating in the Environment and Among Children with Acute Gastroenteritis. J. Biomed. Res. Environ. Sci..

[25] Bouseettine R., Hassou N., Hatib A., Berradi B., Bessi H., Ennaji M.M. (2020). Worldwide Emerging and Reemerging Rotavirus Genotypes: Genetic Variability and Interspecies Transmission in Health and Environment. Emerging and Reemerging Viral Pathogens: Volume 1: Fundamental and Basic Virology Aspects of Human, Animal and Plant Pathogens.

[26] Kirkwood C.D., Ma L.F., Carey M.E., Steele A.D. (2019). The Rotavirus Vaccine Development Pipeline. Vaccine.

[27] Ruiz M.C., Leon T., Díaz Y., Michelangeli F. (2009). Molecular Biology of Rotavirus Entry and Replication. Sci. World J..

[28] Jiang L., Tang A., Song L., Tong Y., Fan H. (2023). Advances in the Development of Antivirals for Rotavirus Infection. Front. Immunol..

[29] de la Flor i Brú J. (2019). Gastroenteritis Aguda. Pediatr. Integral.

[30] Steyer A., Mičetić-Turk D., Fijan S. (2022). The Efficacy of Probiotics as Antiviral Agents for the Treatment of Rotavirus Gastrointestinal Infections in Children: An Updated Overview of Literature. Microorganisms.

[31] Rocha M.P., Amorim J.M., Lima W.G., Brito J.C.M., da Cruz Nizer W.S. (2022). Effect of Honey and Propolis, Compared to Acyclovir, against Herpes Simplex Virus (HSV)-Induced Lesions: A Systematic Review and Meta-Analysis. J. Ethnopharmacol..

[32] Yosri N., Abd El-Wahed A.A., Ghonaim R., Khattab O.M., Sabry A., Ibrahim M.A.A., Moustafa M.F., Guo Z., Zou X., Algethami A.F.M. (2021). Anti-Viral and Immunomodulatory Properties of Propolis: Chemical Diversity, Pharmacological Properties, Preclinical and Clinical Applications, and in Silico Potential against SARS-CoV-2. Foods.

[33] Vilhelmova-Ilieva N.M., Nikolova I.N., Nikolova N.Y., Petrova Z.D., Trepechova M.S., Holechek D.I., Todorova M.M., Topuzova M.G., Ivanov I.G., Tumbarski Y.D. (2023). Antiviral Potential of Specially Selected Bulgarian Propolis Extracts: In Vitro Activity against Structurally Different Viruses. Life.

[34] Saifulazmi N.F., Rohani E.R., Harun S., Bunawan H., Hamezah H.S., Nor Muhammad N.A., Azizan K.A., Ahmed Q.U., Fakurazi S., Mediani A. (2022). A Review with Updated Perspectives on the Antiviral Potentials of Traditional Medicinal Plants and Their Prospects in Antiviral Therapy. Life.

[35] Mendonça R.Z., Nascimento R.M., Fernandes A.C.O., Silva P.I. (2024). Antiviral Action of Aqueous Extracts of Propolis from *Scaptotrigona aff. postica* (Hymenoptera; Apidae) against Zica, Chikungunya, and Mayaro Virus. Sci. Rep..

[36] Rocha V.M., Portela R.W., Lacerda L.E., Sokolonski A.R., de Souza C.O., dos Anjos J.P., Nascimento R.Q., Umsza-Guez M.A. (2024). Propolis from Different Brazilian Stingless Bee Species: Phenolic Composition and Antimicrobial Activity. Food Prod. Process. Nutr..

[37] Escriche I., Juan-Borrás M. (2018). Standardizing the Analysis of Phenolic Profile in Propolis. Food Res. Int..

[38] Superti F., Tinari A., Baldassarri L., Donelli G. (1991). HT-29 Cells: A New Substrate for Rotavirus Growth. Arch. Virol..

[39] Romero-Arguelles R., Tamez-Guerra P., González-Ochoa G., Romo-Sáenz C.I., Gomez-Flores R., Flores-Mendoza L., Aros-Uzarraga E. (2023). Bifidobacterium Longum and Chlorella Sorokiniana Improve the IFN Type I-Mediated Antiviral Response in Rotavirus-Infected Cells. Microorganisms.

[40] Chasey D. (1980). Investigation of Immunoperoxidase-Labelled Rotavirus in Tissue Culture by Light and Electron Microscopy. J. General. Virol..

[41] Alvarez A., Barisone G.A., Diaz E. (2014). Focus Formation: A Cell-Based Assay to Determine the Oncogenic Potential of a Gene. J. Vis. Exp..

[42] (2009). Biological Evaluation of Medical Devices. Part 5: Tests for In Vitro Cytotoxicity.

[43] Barboza J.R., Pereira F.A.N., Fernandes R.A., Vasconcelos C.C., Cartágenes M.d.S.d.S., Oliveira Lopes A.J., Melo A.C.d., Guimarães I.d.S., Rocha C.Q.d., Ribeiro M.N.d.S. (2020). Cytotoxicity and Pro-Apoptotic, Antioxidant and Anti-Inflammatory Activities of Geopropolis Produced by the Stingless Bee *Melipona fasciculata* Smith. Biology.

[44] Choudhari M.K., Haghniaz R., Rajwade J.M., Paknikar K.M. (2013). Anticancer Activity of Indian Stingless Bee Propolis: An In Vitro Study. Evid.-Based Complement. Altern. Med..

[45] Miryan M., Alavinejad P., Abbaspour M., Soleimani D., Ostadrahimi A. (2020). Does Propolis Affect the Quality of Life and Complications in Subjects with Irritable Bowel Syndrome (Diagnosed with Rome IV Criteria)? A Study Protocol of the Randomized, Double-Blinded, Placebo-Controlled Clinical Trial. Trials.

[46] Hossain S., Yousaf M., Liu Y., Chang D., Zhou X. (2022). An Overview of the Evidence and Mechanism of Drug–Herb Interactions Between Propolis and Pharmaceutical Drugs. Front. Pharmacol..

[47] Cheung K.W., Sze D.M.Y., Chan W.K., Deng R.X., Tu W., Chan G.C.F. (2011). Brazilian Green Propolis and Its Constituent, Artepillin C Inhibits Allogeneic Activated Human CD4 T Cells Expansion and Activation. J. Ethnopharmacol..

[48] Suffness M., Pezzuto J. (1990). Assays Related to Cancer Drug Discovery. Methods in Plant Biochemistry: Assays for Bioactivity.

[49] Tutunchi H., Naeini F., Ostadrahimi A., Hosseinzadeh-Attar M.J. (2020). Naringenin, a Flavanone with Antiviral and Anti-Inflammatory Effects: A Promising Treatment Strategy against COVID-19. Phytother. Res..

[50] Cai J., Wen H., Zhou H., Zhang D., Lan D., Liu S., Li C., Dai X., Song T., Wang X. (2023). Naringenin: A Flavanone with Anti-Inflammatory and Anti-Infective Properties. Biomed. Pharmacother..

[51] Banerjee S., Sarkar R., Mukherjee A., Miyoshi S.I., Kitahara K., Halder P., Koley H., Chawla-Sarkar M. (2022). Quercetin, a Flavonoid, Combats Rotavirus Infection by Deactivating Rotavirus-Induced pro-Survival NF-ΚB Pathway. Front. Microbiol..

[52] Choi H.J. (2023). Antiviral Activity of Flavonoids Against Non-Polio Enteroviruses. J. Bacteriol. Virol..

[53] Wang D., Chen J., Pu L., Yu L., Xiong F., Sun L., Yu Q., Cao X., Chen Y., Peng F. (2023). Galangin: A Food-Derived Flavonoid with Therapeutic Potential against a Wide Spectrum of Diseases. Phytother. Res..

[54] Huang H., Liao D., Zhou G., Zhu Z., Cui Y., Pu R. (2020). Antiviral Activities of Resveratrol against Rotavirus in Vitro and in Vivo. Phytomedicine.

[55] Pal D., Bareth K., Rani P., Kandar C.C., Mishra A. (2024). Coumarins as Emerging Anti-Viral Compounds from Natural Origins: Ethnopharmacology, Chemistry, Mechanism of Action, Clinical and Preclinical Studies, and Future Perspectives. Anti-Viral Metabolites from Medicinal Plants.

[56] de Carvalho F.M.d.A., Schneider J.K., de Jesus C.V.F., de Andrade L.N., Amaral R.G., David J.M., Krause L.C., Severino P., Soares C.M.F., Caramão Bastos E. (2020). Brazilian Red Propolis: Extracts Production, Physicochemical Characterization, and Cytotoxicity Profile for Antitumor Activity. Biomolecules.

[57] Arung E.T., Ramadhan R., Khairunnisa B., Amen Y., Matsumoto M., Nagata M., Kusuma I.W., Paramita S., Sukemi, Yadi (2021). Cytotoxicity Effect of Honey, Bee Pollen, and Propolis from Seven Stingless Bees in Some Cancer Cell Lines. Saudi J. Biol. Sci..

[58] Rizwan M., Cheng K., Gang Y., Hou Y., Wang C. (2025). Immunomodulatory Effects of Vitamin D and Zinc on Viral Infection. Biol. Trace Elem. Res..

[59] da Silva Lima F., da Rocha Romero A.B., Hastreiter A., Nogueira-Pedro A., Makiyama E., Colli C., Fock R.A. (2018). An Insight into the Role of Magnesium in the Immunomodulatory Properties of Mesenchymal Stem Cells. J. Nutr. Biochem..

[60] Bilal Hussain M., Hassan S., Waheed M., Javed A., Adil Farooq M., Tahir A. (2019). Bioavailability and Metabolic Pathway of Phenolic Compounds. Plant Physiological Aspects of Phenolic Compounds.

[61] Kyselova Z. (2011). Toxicological Aspects of the Use of Phenolic Compounds in Disease Prevention. Interdiscip. Toxicol..

[62] Catalkaya G., Venema K., Lucini L., Rocchetti G., Delmas D., Daglia M., De Filippis A., Xiao H., Quiles J.L., Xiao J. (2020). Interaction of Dietary Polyphenols and Gut Microbiota: Microbial Metabolism of Polyphenols, Influence on the Gut Microbiota, and Implications on Host Health. Food Front..

[63] Antunes Moura R.T., Bueno N.B., Silva-Neto L.G.R., Pureza I.R.d.O.M., da Silva M.G.V., Cabral M.J., Florêncio T.M.d.M.T. (2022). Red Propolis Supplementation Does Not Decrease Acute Respiratory Events in Stunted Preschool Children: A Paired Nonrandomized Clinical Trial. Clin. Nutr. ESPEN.

[64] MedlinePlus National Library of Medicine Propolis. https://medlineplus.gov/druginfo/natural/390.html.

[65] Kara M., Sütçü M., Kılıç Ö., Gül D., Tural Kara T., Akkoç G., Baktır A., Bozdemir Ş.E., Özgür Gündeşlioğlu Ö., Yıldız F. (2025). Propolis as a Treatment Option for Hand, Foot, and Mouth Disease (HFMD) in Children: A Prospective Randomized Clinical Study. Children.

